# Heart type fatty acid binding protein response and subsequent development of atherosclerosis in insulin resistant polycystic ovary syndrome patients

**DOI:** 10.1186/1757-2215-5-45

**Published:** 2012-12-18

**Authors:** Evrim Cakir, Mustafa Ozbek, Mustafa Sahin, Erman Cakal, Askin Gungunes, Zeynep Ginis, Taner Demirci, Tuncay Delibasi

**Affiliations:** 1 Department of Endocrinology and Metabolic Diseases, Diskapi Training and Research Hospital, Ankara, Turkey; 2 Department of Biochemistry, Diskapi Training and Research Hospital, Ankara, Turkey; 3 Yeni Ziraat mahallesi, 660. sokak, Kanarya Apt. no : 10/ 4, TR-06200, Altındağ, Ankara, Turkey

**Keywords:** Polycystic ovary syndrome, Heart type free fatty acid binding protein, Carotid intima media thickness, Insulin resistance, Cardiovascular disease risk

## Abstract

**Background:**

Women with polycystic ovary syndrome (PCOS) have higher risk for cardiovascular disease (CVD). Heart type fatty acid binding protein (HFABP) has been found to be predictive for myocardial ischemia.Wet ested whether HFABP is the predictor for CVD in PCOS patients, who have an increased risk of cardiovascular disease.

**Methods:**

This was a prospective, cross sectional controlled study conducted in a training and research hospital.The study population consisted of 46 reproductive-age PCOS women and 28 control subjects. We evaluated anthropometric and metabolic parameters, carotid intima media thickness and HFABP levels in both PCOS patients and control group.

**Results:**

Mean fasting insulin, homeostasis model assessment insulin resistance index (HOMA-IR), triglyceride, total cholesterol, low density lipoprotein cholesterol, free testosterone, total testosterone, carotid intima media thickness (CIMT) levels were significantly higher in PCOS patients. Although HFABP levels were higher in PCOS patients, the difference did not reach statistically significant in early age groups. After adjustment for age and body mass index, HFABP level was positive correlated with hsCRP, free testosterone levels, CIMT and HOMA-IR.

**Conclusions:**

Heart type free fatty acid binding protein appeared to have an important role in metabolic response and subsequent development of atherosclerosis in insulin resistant, hyperandrogenemic PCOS patients.

## Introduction

Polycystic ovary syndrome (PCOS) is a common endocrine disorder affecting at least five to10% of women of reproductive age [1]. Polycystic ovary syndrome is characterized by hyperandrogenism, menstrual disturbance, anovulation, infertility and obesity [2], and also associated with an increased number of cardiovascular risk factors [3], and early atherosclerosis [4,5]. Hyperinsulinism and insulin resistance are frequent findings in PCOS patients, and these traits have cause-consequence relationships with low-grade chronic inflammation [6], and increased cardiovascular disease (CVD) risk [7].

Heart-type fatty acid-binding protein (H-FABP) is a low molecular-weight cytoplasmic protein that is abundant in the myocardium, and provides intracelluler uptake of free fatty acid protein [8].

Several studies have assessed free fatty acid binding protein (FABP) and shown to positively correlated with cardiometabolic risk factors [9-11], but heart type free acid binding protein has not been evaluated in PCOS patients, yet.

The aim of this study was to evaluate the H-FABP in PCOS patients and its association between cardiometabolic factors.

## Materials and methods

We studied 46 patients with PCOS and age- body mass index (BMI) matched 28 healthy controls consisting of women with regular ovulatory cycles and normal androgen levels. All patients gave a written consent. All patients were female and nonsmokers.

The diagnosis of PCOS was made according to the Rotterdam European Society for Human reproduction and Embryology/ American Society for Reproductive Medicine–sponsored PCOS Consensus Workshop Group [12]. The revised diagnostic criteria of PCOS is as follows, with at least two of the following being required;

1. Oligo and/or anovulation that is menstrual disturbance

2. Clinical and/or biochemical signs of hyperandrogenism

3. Polycystic ovarian appearance on ultrasound

Participants who had smoking history, diabetes mellitus, hyperprolactinemia, congenital adrenal hyperplasia, androgen-secreting tumours, thyroid disorders, Cushing syndrome (1 mg dexamethasone suppression test), infection diseases, hypertension, hepatic or renal dysfunction were excluded from the study. Patients were also excluded if they had used within 3 months before enrollment confounding medications, including oral contraceptive agents, antilipidemic drugs, hypertensive medications, and insulin-sensitizing drugs.

Control group (n = 28) consisted of healthy patients who were admitted to check-up unit without any systemic disorder. All of the women in the control group had hirsutism score <8. All women in the control group had regular menses, every 21–35 days. None of the women in the control group had polycystic ovary in ultrasound.

Weight and height were measured in light clothing without shoes. BMI was calculated, dividing the weight divided by square of height (kg/m^2^). Waist circumference was measured at the narrowest level between the costal margin and iliac crest, and the hip circumference was measured at the widest level over the buttocks while the subjects were standing and breathing normally. The waist-to-hip ratio (WHR) was calculated.

The degree of hirsutism was determined by Ferriman-Gallwey (FG) scoring [13]. The BMI, WHR and hirsutism scores were assessed by a single investigator for all of the subjects.

### Measurement of carotid intima media thickness

Carotid intima media thickness (CIMT) was derived from noninvasive ultrasound of the common carotid arteries, using a high-resolution ultrasound machine (Sonoline G 40, Siemens) with 7.5 MHz mechanical sector transducer. The intima media thickness was defined as the distance between the blood-intima and media-adventitia boundaries on B-mode imaging.

#### Biochemical evaluation

Venous blood samples were obtained in the follicular phase of a spontaneous or progesterone induced menstrual cycle. Before the study, blood samples were drawn from each patient after 12 h overnight fasting for the determination of hormones, lipid profile, high-sensitive C- reactive protein (hs-CRP), insulin levels, glucose levels.

Plasma glucose was determined with glucose oxidase/peroxidase method (Gordion Diagnostic, Ankara, Turkey). Serum levels of follicle-stimulating hormone (FSH), luteinizing hormone (LH), prolactin, dehydroepiandrosterone sulfate (DHEAS), total testosterone (T), insulin and thyroid stimulating hormone (TSH) were measured with specific electrochemiluminescence immunoassays (Elecsys 2010 Cobas, Roche Diagnostics, Mannheim, Germany). Serum 17 hydroxyprogesterone was measured by radioimmunoassay. Levels of total-cholesterol, high density lipoprotein cholesterol (HDL-C), and triglyceride (TG) were determined with enzymatic colorimetric assays by spectrophotometry (BioSystems S.A., Barcelona, Spain). Low density lipoprotein cholesterol (LDL-C) was calculated using the Friedewald formula.

Serum hs-CRP was determined using high-sensitive CRP immunonephelometry (BN, Dade-Behring; Marburg, Germany). The cut off for hsCRP was taken 1.5 [14].

Insulin resistance was calculated by using the homeostasis model assessment insulin resistance index (HOMA-IR) [15], according to the formula, fasting plasma glucose (mmol/L) x fasting serum insulin (mU/mL) /22.5. The cut off value was taken 2.7 for HOMA-IR [16].

### Heart-type fatty acid binding protein

The human H-FABP ELISA is a ready-to-use solidphase enzyme-linked immunosorbent assay based on the sandwich principle. Samples and standards are incubated together with peroxidase-conjugated second antibody in microtiter wells coated with antibodies recognizing human H-FABP. During incubation human H-FABP is captured by the solid bound antibody. The secondary antibodies will bind to the captured human H-FABP. The peroxidase conjugated antibody will react with the substrate, tetramethylbenzidine (TMB). The enzyme reaction is stopped by the addition of oxalic acid. The absorbance at 450 nm is measured with a spectrophotometer.

#### Statistical analyses

Collected data was entered to SPSS version 17. Continuous data were shown as mean ± SD. Chi-squared tests were used to compare differences in rates. Normally distributed variables were compared by using Student T test. Data that were not normally distributed, as determined using Kolmogorov–Smirnov test, were logarithmically transformed before analysis. Data are expressed as mean ± SD or median with interquartile range as appropriate. Degree of association between continuous variables was calculated by Pearson correlation coefficient, nonnormally distributed variables was evaluated by spearman’s rho correlation coefficient. The multiple linear regression enter method was used to determine the independent predictors. Binary logistic regression analysis was used to calculate odds ratio and roc curve analysis was used to determine cut off value for HFABP with optimal sensitivity and specificity. Univariate analyses were used to adjust HFABP with respect to age, BMI.

p value lower than 0.05 was accepted as statistically significant.

## Results

Clinical and endocrinological parameters screened in patients with PCOS and in healthy control subjects were shown in Table 1. We studied 46 PCOS patients (21.97 ± 4.99 mean age, range 18–33 years; BMI; 24.5 ± 5.51 kg /m^2^) and 28 age and BMI matched healthy control group (23.42 ± 4.7 mean age, range 18–32 years, BMI; 23.77 ± 4.71 kg/m^2^).

**Table 1:** The clinical and biochemical / hormonal data in women with polycystic ovary syndrome (PCOS) patients and healthy controls

**Variable**	**Women with PCOS (n _ 46)**	**Healthy controls (n _ 28)**	***P***
Age, years	21.97 ± 4.19	23.42 ± 4.70	0.17
BMI, kg/m2	24.50 ± 5.51	23.77 ± 4.91	0.56
Waist/hip ratio	0.81 ± 0.73	0.81 ± 0.63	0.97
Fasting insulin, μ IU/ml	13.84(4.03-47.97)	10.35(2.1-25.8)	0.007
Fasting glucose, mg/dl	91.23 ± 8.24	89.07 ± 9.85	0.31
HOMA-IR	3.21(0.38-12.25)	2.23(0.47-6.43)	0.011
Total cholesterol, mg/dl	173.63 ± 29.41	154.46 ± 26.74	0.006
Triglyceride, mg/dl	111.65 ± 52.80	78.53 ± 30.30	0.002
HDL-C, mg/dl	51.47 ± 12.36	50.78 ± 11.68	0.81
LDL-C, mg/dl	99.68 ± 26.60	87.62 ± 22.58	0.04
hsCRP, mg/L	1.33(0.15-15.8)	0.97(0.15-9)	0.35
TSH, μ IU/ml	2.19 ± 0.96	1.72 ± 0.81	0,03
FSH, m IU/ml	5.49 ± 1.71	5.98 ± 1.86	0.25
LH, m IU/ml	5.69 ± 1.84	5.63 ± 2.25	0.90
Estradiol, pg/ml	40.68 ± 23.25	71.12 ± 42.00	<0.001
Free testosterone,pg/ml	2.99 ± 1.09	1.58 ± 0.51	<0.001
Total testosterone, ng/ml	0.56 ± 0.27	0.41 ± 0.14	0.01
17-OHprogesterone,ng/ml	1.45 ± 0.60	0.98 ± 0.57	0.002
DHEAS, μq/dl	292.60 ± 138.54	175.28 ± 81.63	<0.001
CIMT, mm	0.42 ± 0.056	0.37 ± 0.061	<0,001
HFABP, ng/dl	39.87(22.88-135.12)	35.64(17.86- 79.97)	0.419

*BMI* body mass index, *HOMA-IR* homeostasis model assessment insulin resistance index, *HDL-C* high density lipoprotein cholesterol, *LDL-C* LOW density lipoprotein cholesterol, *hs-CRP* high-sensitive C- reactive protein, *TSH* thyroid stimulating hormone, *FSH* follicle-stimulating hormone, *LH* luteinizing hormone, *DHEAS* dehydroepiandrosterone sulfate, *CIMT* carotid intima media thickness, *HFABP* heart fatty acid binding protein.

Mean fasting insulin, HOMA-IR, triglyceride, total cholesterol, LDL-C, free testosterone, total testosterone, 17 OH progesterone, DHEAS, CIMT levels were significantly higher and estradiol were significantly lower in PCOS patients (p < 0.05) (Table 1).

**Figure 1:**
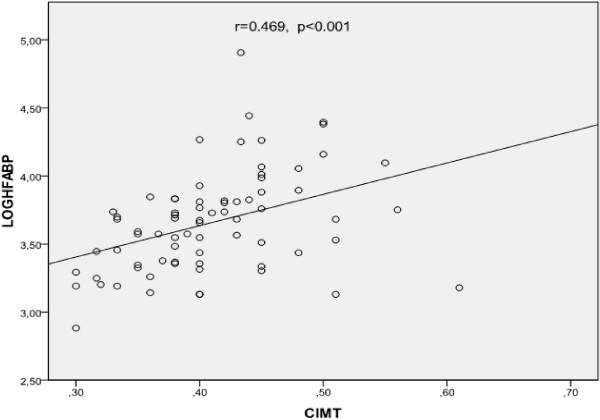
Correlation between log transformed H-FABP and carotid intima media thickness.

Mean HFABP level was 42.40 ± 19.08 ng/dl in PCOS patients while mean HFABP level was 40.52 ± 17.43 ng/dl in healthy control women (p < 0.67). Although mean HFABP level was higher in PCOS patients the difference did not reach statistical significance. After adjustment for age and BMI, HFABP level was positive correlated with FG score, fasting glucose, fasting insulin, TG, TSH, hsCRP, DHEAS, free testosterone levels, CIMT, HOMA-IR and negative correlated with HDL-C and estradiol levels (Table 2). In multiple linear regression analyses logarithmic transformed HFABP was found to be significantly associated with CIMT (beta coefficient = 0.389, p = 0.002) (age, BMI were included in the model). The correlation between log transformed HFABP and CIMT was shown in Figure 1.

**Table 2:** Correlation of Age and BMI Adjusted HFABP levels with cardiometabolic parameters

**Variable**	**r**	***P***
FG**	0.341	0.003
Fasting glucose**	0.333	0.004
Fasting insulin**	0.677^a^	< 0.001
HOMA**	0.720^a^	<0.001
TG**	0.286	0.013
HDL-C**	−0.424	<0.001
DHEAS*	0.233	0.045
Free Testosterone*	0.232	0.047
Estradiol*	−0.257	0.027
TSH**	0.335	0.003
CIMT**	0.468	< 0.001
hsCRP**	0.494 ^a^	<0.001

r indicates Spearman’s rho correlation coefficient; *Correlation is significant at the 0.05 level. **Correlation is significant at the 0.01 level. ^a^ log transformed.

*HFABP* heart fatty acid binding protein, *FG* Ferriman Gallwey Score, *HOMA* homeostasis model assessment insulin resistance index, *TG* triglyceride, *HDL-C* High density lipoprotein cholesterol, *DHEAS* dehydroepiandrosterone sulfate, *TSH* thyroid stimulating hormone, *CIMT* Carotid intima media thickness, *hsCRP* high sensitivity C-reactive protein.

Mean CIMT level was 0.42 ± 0.056 millimeter in PCOS patients while mean CIMT level was 0.37 ± 0.061 millimeter in healthy control women (p < 0.001). A significant positive correlation was found between HFABP, BMI, FG, fasting glucose, triglyceride, PRL, free testosterone, HOMA-IR, fasting insulin, hs-CRP and CIMT measurement (Table 3).

**Table 3:** Correlation of Carotid Intima Media Thickness with Cardiometabolic Parameters

**Variable**	**r**	***P***
BMI **	0.408	< 0.001
FG**	0.390	0.001
Fasting glucose*	0.283	0.014
Fasting insulin*	0.267	0.021
HOMA*	0.294	0.011
TG**	0.316	0.006
Free Testosterone*	0.246	0.035
HFABP**	0.396	<0.001
hsCRP*	0.293	0.011

*r* indicates Pearson correlation coefficient, *BMI* body mass index, *FG* ferriman gallwey score, *HOMA* homeostasis model assessment insulin resistance index, *TG* triglyceride, *HFABP* heart fatty acid binding protein, *hsCRP* high sensitivity C-reactive protein.

*Correlation is significant at the 0.05 level. **Correlation is significant at the 0.01 level.

**Figure 2:**
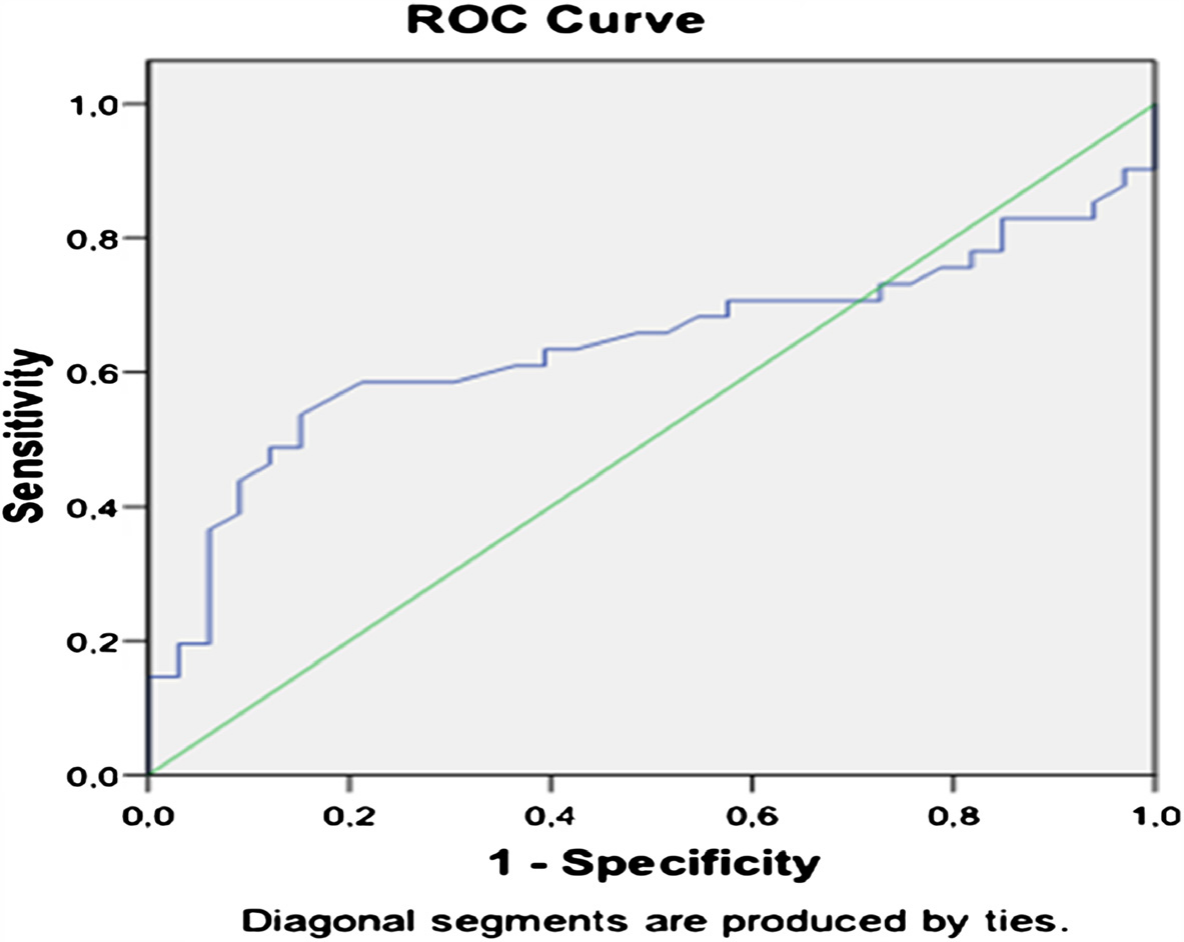
**The predictive performance of heart type free fatty acid binding protein for high homeostasis model assessment –insulin resistance index according to the receiver operating characteristic (ROC) curve.** AUC; area under the sensitivity-specificity curve, 95% CI; 95% confidence interval.

In ROC curve analysis HOMA-IR ≥2.7 can be predicted by the use of HFABP at a cut off value of 37.51 with 65%sensitivity and 61% specificity (area under curve 0.654, 95% confidence interval 0.52 to 0.78, p = 0.022) (Figure 2). In binary logistic regression analyses HFABP level higher than 37.51 ng/dl was risk factor for higher HOMA-IR index (OR: 3,022 95% CI(1,17-7,79), p = 0.022).

There were 29 (63%) patients with high HOMA-IR in PCOS group, 9 (32.1%) in control (p = 0.01); 26(56.5%) patients had high hsCRP levels in PCOS group, 12(42.9%) in control (p = 0.254).

The participants were divided into 2 groups according to hsCRP levels. The participants whose hsCRP levels higher than 1.5 mg/L had higher HFABP levels but this difference did not reach statistically significance. Also, HFABP levels were higher in group with high FG score (FG score ≥8). Heart type FABP protein was found statistically significant higher in participants with high HOMA-IR group (Table 4).

**Table 4:** The comparison of CIMT and HFABP in FG, HOMA, hsCRP groups

**Variable**	**CIMT*Mean*** **± SD**	***P***	**HFABP*Median(Min-Max)***	***P***
**FG**				
<8	0.38 ± 0.06	0.006	35.64(17.8-135.1)	0.232
≥8	0.42 ± 0.05		41.9(22.8-85.0)	
**HOMA**				
<2.7	0.38 ± 0.05	<0.001	35.03(23.1-70.2)	0.022
≥2.7	0.43 ± 0.06		43.88(22.8-135.1)	
**hsCRP**				
<1.5	0.38 ± 0.05	0.001	35.03(17.8-71.3)	0.071
≥1.5	0.43 ± 0.06		41.78(22.8-135.1)	

## Discussion

The results of this study demonstrate that the mean H-FABP level was similar in early age of PCOS patients and control group. Heart type FABP showed significant correlations with cardiometabolic parameters independent of age and obesity.

In recent substudy of the Women’s Ischemia Evaluation Study (WISE) (14) shown that women with PCOS have a larger number of cardiovascular events. In this study, CVD was positively correlated with free testosterone. In addition, the event free survival (including fatal and nonfatal events) was significantly lower in PCOS compared with non-PCOS women. In our study cardiometabolic parameters including HOMA-IR, TG, LDL-C, free testosterone, DHEAS, CIMT were significantly higher in PCOS patients and positively correlated with HFABP. Also, FG that reflects androgen effects was positively correlated with HFABP and CIMT.

In Victor et al. study an association was found between insulin resistance and an impaired endothelial and mitochondrial oxidative metabolism. They concluded that the inflammatory state related to insulin resistance in PCOS affects endothelial function [17]. In presented study hsCRP and insulin resistance were found positive correlated with CIMT consistent with this hypothesis.

The HFABP isoform is produced by cardiomyocytes, skeletal muscle [18], kidney distaltubular cells [19], and specific parts of the brain [20]. Free acid binding protein provides intracellular translocation of long-chain fatty acids [21]. Heart type FABP contributes signal transduction pathways [22], and is protective for cardiac myocytes against the high concentrations of long-chain fatty acids [21,23].

The plasma concentration of HFABP is influenced by a variety of factors such as cytosolic enzymes, subcellular location, molecular mass and concentration gradients. Additionally, plasma clearance of FABP also affects the appearance of its level in the general circulation. Because of its smaller size, FABP pass through the glomerular membranes and is reabsorbed and metabolized in tubular epithelial cells [24]. So, the impaired clearance due to renal failure can cause falsely increased plasma concentrations, on contrary hypermetabolic states can cause falsely decreased levels of FABP [25].

The hypothesis suggest that the release of HFABP is a metabolically controlled property of cell membranes due to reversible disturbance of cell metabolism [26]. Increased plasma H-FABP concentrations significantly correlated with increased cardiac event rates and cardiac mortality [27,28].

A growing evidence has been found that adipocyte type FABP (AFABP) has an great role in atherosclerotic process and metabolic risk factors [9,29-32]. A population based study on long-term follow-up, subjects with higher baseline AFABP levels had progressively worse cardiometabolic risk profile and increasing risk of the MetS [10].

A-FABP has been found predictive for MetS even after adjustment for its individual components whereas there are still few studies on the association between cardiometabolic parameters and HFABP.

Heart type FABP has been shown to be a more sensitive early marker for identification of acute myocardial injury [33,34]. In recent study HFABP has been found significantly higher in patients with acromegaly than in control subjects [35]. In another study H-FABP levels were also found significantly higher in patients with MetS than in control subject [11]. In Yan et al. study HFABP has been found a useful marker for illustrate organ dysfunction and leptin has been shown to reduce sepsis-induced organ injuries by restraining HFABP tissue levels in the mouse model of sepsis [36]. However, in PCOS patients HFABP level has not been evaluated, yet. In our study we evaluated HFABP level in PCOS patients and we observed PCOS patients had higher HFABP level but the difference did not reach statistically significance in our early age group. Additionally, we obtained positive correlation between HFABP and CIMT.

Heart type FABP may have a predictive role for detecting cardiometabolic risk in potential diseases. In early age of PCOS patients the HFABP levels are similar to control, it is thought to be due to high metabolic rate and short disease duration in early age group. However its higher level has been found to associated with inflammation and cardiometabolic risk. Therefore H-FABP seems to be a marker that will enable the detection of cardiac injury in advancing age PCOS patients at an early stage.

Heart type FABP appeared to have an important role in metabolic response and subsequent development of atherosclerosis in insulin resistant, hyperandrogenemic PCOS patients.

## Competing interests

The authors declare that they have no competing interests.

## Authors’ contribution

EC: have made contributions to conception and design, acquisition of data, and analysis and interpretation of data. MO: have made contributions to conception and design, acquisition of data, and analysis and interpretation of data MS: have made contributions to conception and design, acquisition of data, and analysis and interpretation of data. EC: have made contributions to conception and design, acquisition of data, and analysis and interpretation of data TD: have made contributions to acquisition of data. ZG: have made contributions to measure of collected blood. AG:have made contributions to acquisition of data. TD: have made contributions to conception, design and interpretation of data. All authors read and approved the final manuscript.
